# Isolation and characterization of *Burkholderia* sp. strain CCA53 exhibiting ligninolytic potential

**DOI:** 10.1186/s40064-016-2237-y

**Published:** 2016-05-11

**Authors:** Hironaga Akita, Zen-ichiro Kimura, Mohd Zulkhairi Mohd Yusoff, Nobutaka Nakashima, Tamotsu Hoshino

**Affiliations:** Research Institute for Sustainable Chemistry, National Institute of Advanced Industrial Sciences and Technology (AIST), 3-11-32 Kagamiyama, Higashi-Hiroshima, Hiroshima 739-0046 Japan; Department of Civil and Environmental Engineering, National Institute of Technology, Kure College, 2-2-11 Aga-minami, Kure, Hiroshima 737-8506 Japan; Department of Bioprocess Technology, Faculty of Biotechnology and Biomolecular Sciences, Universiti Putra Malaysia, 43400 Serdang, Selangor Malaysia; Bioproduction Research Institute, National Institute of Advanced Industrial Sciences and Technology (AIST), 2-17-2-1 Tsukisamu-Higashi, Toyohira-ku, Sapporo, 062-8517 Japan; Department of Biological Information, Graduate School of Bioscience and Biotechnology, Tokyo Institute of Technology, 2-12-1-M6-5 Ookayama, Meguro-ku, Tokyo, 152-8550 Japan

**Keywords:** 16S rRNA gene sequencing, *Burkholderia*, Lignin-associated aromatic monomer, Lignin-degrading bacterium

## Abstract

Microbial degradation of lignin releases fermentable sugars, effective utilization of which could support biofuel production from lignocellulosic biomass. In the present study, a lignin-degrading bacterium was isolated from leaf soil and identified as *Burkholderia* sp. based on 16S rRNA gene sequencing. This strain was named CCA53, and its lignin-degrading capability was assessed by observing its growth on medium containing alkali lignin or lignin-associated aromatic monomers as the sole carbon source. Alkali lignin and at least eight lignin-associated aromatic monomers supported growth of this strain, and the most effective utilization was observed for *p*-hydroxybenzene monomers. These findings indicate that *Burkholderia* sp. strain CCA53 has fragmentary activity for lignin degradation.

## Background

One of the consequences of the increasing population and industrial development is greater fossil fuel consumption and, in turn, greenhouse gas emission, which exacerbates the problem of global warming. It has been proposed that use of biofuels produced from biomass feedstocks could serve as alternatives to fossil fuel that would slow global warming. First-generation biofuels are mostly produced from edible feedstocks such as corn, sugarcane and starch (Ho et al. 2014; Islam et al. 2015). The advantages of edible feedstocks are that they have a high sugar content, require simple sugar extraction methods, and a variety of technologies for biofuel production are available. However, edible feedstocks are also consumed as our food and by our livestock, which means biofuel production competes with production to meet our dietary needs. By contrast, second-generation biofuels are produced from lignocellulosic biomass, which is usually indigestible (Ho et al. 2014; Islam et al. 2015). Moreover, lignocellulosic biomass is widely distributed and is the most abundantly available raw material on Earth.

Unfortunately, industrial production of second-generation biofuels faces significant obstacles, as the production process is much more laborious than that of first-generation biofuels. The microbial process for converting lignocellulosic biomass to biofuel typically consists of three steps: pretreatment, enzymatic hydrolysis and fermentation. At the pretreatment step, cellulose, hemicellulose and lignin are released through decomposition of the complex structure of lignocellulosic biomass. At the enzymatic hydrolysis step, cellulosic and hemicellulosic polysaccharides are converted into saccharified solution containing mixed sugars. Finally, the saccharified solution is fed to microorganisms as a carbon source in the fermentation step (Ho et al. 2014; Islam et al. 2015). However, lignin is composed of heterogeneous aromatic acids that commonly used industrial host microorganisms such as *Saccharomyces cerevisiae* and *Escherichia coli* cannot efficiently assimilate. On the contrary, several aromatic acids produced by lignin decomposition cause membrane disruption, enzyme inhibition and DNA damage in microbial cells, ultimately reducing productivity. A few bacteria, including *Phanerochaete chrysosporium*, *Rhodococcus erythropoli*s and *Streptomyces coelicolor*, are known to assimilate lignin (Ahmad et al. 2010). However, these microorganisms are not suitable for industrial production of second-generation biofuels, because their culture conditions are intricate and their growth are relatively slow. Here we report screening, isolation and characterization of a lignin-degrading bacterium from leaf soil sample. This bacterium exhibited rapid growth on solid medium containing alkali lignin as the sole carbon source and assimilated lignin-associated aromatic monomers.

## Methods

### Isolation of the bacterium strain

Soil samples were collected from Higashi-Hiroshima City in Hiroshima Prefecture, Japan. An M9 plate (pH 7.2) was used for isolation and contained the following ingredients: 17 g L^−1^ Na_2_HPO_4_·12H_2_O, 3 g L^−1^ KH_2_PO_4_, 0.5 g L^−1^ NaCl, 1 g L^−1^ NH_4_Cl, 0.24 g L^−1^ MgSO_4_·7H_2_O, 0.011 g L^−1^ CaCl_2_·2H_2_O, 15 g L^−1^ agar and 1 g L^−1^ alkali lignin (Tokyo Chemical Industry, Tokyo, Japan). The plate was inoculated with 1 mL of a 10 % soil wash solution (w/v) and allowed to grow at 37 °C for 2 days. After this initial cultivation, a single colony was successively re-streaked on a new M9 plate at least three times to obtain a pure colony. The purified strain was then grown aerobically at 37 °C in Nutrient Broth (Kyokuto, Tokyo, Japan) and preserved at −20 °C as a suspension in Nutrient Broth supplemented with glycerol (20 %, w/v).

## 16S rRNA gene amplification and sequencing

Genomic DNA from the isolated microorganism was extracted using an illustra bacteria genomicPrep Mini Spin Kit according to manufacturer’s instructions (GE Healthcare, Buckinghamshire, UK) and then used as the template for 16S rRNA gene amplification. 16S rRNA gene was amplified using KOD-plus-DNA polymerase (TOYOBO, Osaka, Japan) with bacterial universal primers 27f (Lane 1991) and 1391r (Turner et al. 1999). After purifying the amplified PCR products using Wizard SV Gel and PCR Clean-Up System (Promega, Madison, WI, USA), they were cloned into pTA2 vector (TOYOBO).

Sequencing was performed using universal primers M13 forward (5′-GTAAAACGACGGCCAGT-3′) and M13 reverse (5′-CAGGAAACAGCTATGAC-3′) on an Applied Biosystems model 3730xl DNA Analyzer at Fasmac (Kanagawa, Japan).

### Characterization of ligninolytic activity

The ligninolytic activity of the isolated microorganism was assessed by monitoring the increase in OD_600_, which reflected the cell growth. The OD_600_ was measured by monitoring the difference between the cell and cell-free turbidity values using an Eppendorf BioSpectrometer (Eppendorf, Hamburg, Germany). The isolated microorganism was pregrown overnight using Nutrient Broth (Kyokuto, Tokyo, Japan) and then diluted 3:100 with fresh M9 medium (pH 7.2) containing 1 g L^−1^ alkali lignin or 5 mM lignin-associated aromatic monomers.

## Results and discussion

### Screening of lignin-degrading bacteria

Microbial degradation of lignin has been primarily studied in white-rot fungi, which are capable of producing several extracellular ligninolytic enzymes, including laccase, lignin peroxidase, manganese peroxidase and versatile peroxidase (Dashtban et al. 2010). Brown-rot fungi are also known to degrade lignin using Fenton-based free radicals (Arantes et al. 2012). Bacterial degradation of lignin has been studied much less (Ahmad et al. 2010; Bugg et al. 2011). A few bacterial species belonging to the genera *Arthrobacter*, *Pseudomonas*, *Sphingobium*, *Streptomyces* and *Rhodococcus* have been shown to have ligninolytic activity in vitro (Bugg et al. 2011). Although these species have several favorable features, including high growth potential, amenability to well developed genetic techniques and ease of practical utilization, their lignin degradation capabilities are much lower than those of fungi. We therefore screened for lignin-degrading bacteria with a high capacity for lignin degradation.

**Fig. 1:**
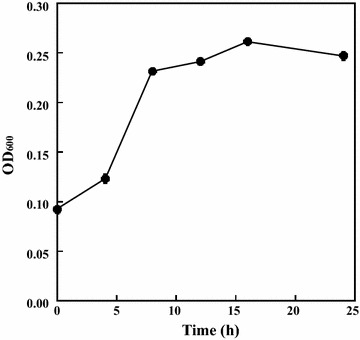
Growth of *Burkholderia* sp. strain CCA53. Culture was performed in M9 liquid medium containing alkali lignin as the sole carbon source. *Error bars* indicate SE (*n* = 3)

To obtain lignin-degrading bacteria, filtrates were prepared from several soil samples and then plated onto M9 plates (pH 7.2) containing alkali lignin as the sole carbon source. When the plate was incubated aerobically at 37 °C, individual colonies were obtained from the leaf soil filtrate. However, except a single colony, individual colonies were not obtained after standard dilution plating onto another M9 plate. Thus, the single colony was obtained and named strain CCA53. When cultured in M9 liquid medium, strain CCA53 exhibited cell growth that depended on lignin assimilation (Fig. 1); a two-fold increase in OD_600_ was observed over the first 8 h, but it remained unchanged thereafter. On the other hand, strain CCA53 showed no-growth in the absence of alkali lignin.

### Phylogenetic analysis

**Fig. 2:**
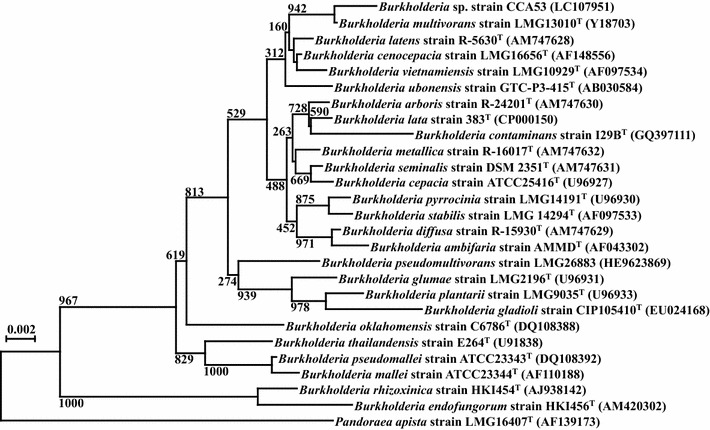
Phylogenetic tree reconstruction based on 16S rRNA gene sequences. All sequences were compared with reference 16S rRNA gene sequences available in the GenBank/EMBL/DDBJ databases using BLAST. Multiple alignment and construction of neighbor-joining phylogenetic tree (Saitou and Nei 1987) were performed using CLUSTAL W version 1.83 (Thompson et al. 1994)

To identify the phylogeny of strain CCA53, the 16S rRNA gene sequence was determined (1303 bp; accession number: LC107951). On the basis of 16S rRNA gene sequence homology, the closest relatives were determined to be *Burkholderia multivorans* strain LMG 13010^T^ (99.7 %), *B. latens* strain R-5630^T^ (99.4 %), *B. cenocepacia* strain LMG 16656^T^ (99.2 %), and with somewhat lower sequence homology, *Pandoraea apista* strain LMG 16407^T^ (95.1 %). In the neighbor-joining phylogenetic tree (Fig. 2), strain CCA53 fell inside the cluster comprising members of the genus *Burkholderia*. Thus, strain CCA53 was identified as *Burkholderia* sp. (strain number: HUT-8135).

*Burkholderia* is a genus of the class β-proteobacteria and is separated from the former *Pseudomonas* rRNA gene homology group II (Yabuuchi et al. 1992). This genus was originally proposed by Yabuuchi et al. (1992) and more than 80 *Burkholderia* species have been reported to date. *Burkholderia* species have been classified into two major clusters and several subgroups through phylogenetic analyses of the 16S rRNA gene, *recA*, *gyrB*, *rpoB* and *acdS* gene sequences as well as their genome sequences (Estrada-de los Santos et al. 2013). Group A comprises plant-associated species and saprophytic species. For example, *B. mimosarum*, *B. nodosa*, *B. sabiae*, *B. tuberum* and *B. phymatum* can facilitate nitrogen fixation in legumes (Suárez-Moreno et al. 2012), and *B. phytofirmans* and *B. unamae* are able to promote plant growth via the activity of 1-aminocyclopropane-1-carboxylate deaminase (Suárez-Moreno et al. 2012). In addition, *B. kururiensis*, *B. phenoliruptrix*, *B. sartisoli*, *B. unamae* and *B. xenovorans* all exhibit abilities to degrade several lignin-associated aromatic monomers and are used for decontamination of polluted soils (Suárez-Moreno et al. 2012), as well as for agricultural purposes such as phytoremediation and biocontrol. On the other hand, group B contains opportunistic pathogens that infect animals, humans and plants. When the phylogenetic relationships of *B. multivorans*, *B. latens* and *B. cenocepacia* were confirmed, their closest relatives were included in group B. Indeed, *B. multivorans*, *B. latens* and *B. cenocepacia* all have an ability to infect cystic fibrosis patients, which leads to pneumonic illness with fever and respiratory failure (Horsley et al. 2011; Jones et al. 2004). However, these species were recently used as biocatalysts for degradation of aromatic polycyclic hydrocarbons, esters and steroid analogs. Non-aqueous phase liquids containing naphthalene, *n*-hexadecane, *n*-octadecane, *n*-nonadecane, 1-methylnaphthalene and pyrene are degraded by *B. multivorans* (NG1) with Triton X-100 or rhamnolipid JBR-515 (Mohanty and Mukherji 2013). Two kinds of *p*-hydroxybenzoic acid esters, methyl paraben and propyl paraben, are degraded by *B. latens* (Amin et al. 2010). Ezetimibe, a selective inhibitor of acyl-coenzyme A, was produced from reduction of 1-(4-fluorophenyl)-5-(2-oxo-4-phenyl-oxazolidin-3-yl)-pentane-1,5-dione in culture medium with *B. cenocepacia* (Singh et al. 2009). It was thus anticipated that *Burkholderia* sp. strain CCA53 would have the capacity to degrade aromatic acids.

### Utilization of lignin-associated aromatic monomers

The main building blocks of lignin are *p*-hydroxybenzene, guaiacyl (4-alkyl-2-methoxyphenol) and syringyl (4-alkyl-2,5-dimethoxyphenol) units, which are crosslinked by C–C bonds (e.g., 5–5, β–1, β–5, β–β) and C–O–C bonds (e.g., 4–O–5, α–O–4, β–O–4) (Vanholme et al. 2010; Zhu et al. 2014). In particular, the β–O–4 bond is the most frequent inter-unit linkage, accounting for more than 50 % of all linkages (Santos et al. 2013).

**Fig. 3:**
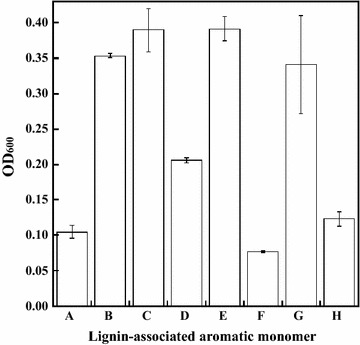
Growth of *Burkholderia* sp. strain CCA53 on lignin-associated aromatic monomers. Growth of *Burkholderia* sp. strain CCA53 in M9 medium containing 5 mM lignin-associated aromatic monomer as the sole carbon and energy source. Experiments were performed in triplicate; *A* Benzaldehyde, *B* Benzoic acid, *C* Catechol, *D* 4-Hydroxy benzaldehyde, *E* 4-Hydroxy benzoic acid, *F* 4-Hydroxybenzyl alcohol, *G* Syringol, *H* Vanillin. *Error bars* indicate SE (*n* = 3)

As described above, when cultured using alkali lignin as sole carbon source, *Burkholderia* sp. strain CCA53 grew inefficiently (Fig. 1). This indicates that this strain has little ability to depolymerize lignin, but it was expected to have an alternative capacity to utilize lignin degradation products. Therefore, to further evaluate the lignin-degrading activity of *Burkholderia* sp. strain CCA53, we assessed its ability to assimilate lignin-associated aromatic monomers. We found that *Burkholderia* sp. strain CCA53 assimilated eight lignin-associated aromatic monomers (Fig. 3). In particular, strong growth improvements were observed with *p*-hydroxybenzene monomers such as 4-hydroxybenzaldehyde and 4-hydroxybenzoic acid. Benzaldehyde, benzoic acid, catechol and vanillin were also assimilated. By contrast, anisole, *o*-cresol, guaiacol, phenol, syringaldehyde, syringic acid, vanillic acid, vanillyl alcohol and veratryl alcohol were not assimilated. Although *Burkholderia* sp. strain CCA53 was able to utilize *p*-hydroxybenzene units, only inefficient growth was observed when lignin was fed as a sole carbon source (Fig. 1). These results imply that *Burkholderia* sp. strain CCA53 lacks activities needed to decompose crosslinks between the main building blocks and to utilize guaiacyl and syringyl units. Consequently, *Burkholderia* sp. strain CCA53 showed inefficient growth.

Several other *Burkholderia* species have also shown lignin-degrading capability (Bugg et al. 2011; Kato et al. 1998; Woo et al. 2014). For example, *B. cepacia* KK01 showed an ability to efficiently assimilate the lignin-associated aromatic monomers *o*-cresol, guaiacol, phenol and vanillic acid, but the assimilation mechanisms have been largely unexplored (Kato et al. 1998). In addition, evidence indicates that the unique lignin-degrading capability of *Burkholderia* sp. strain LIG30 reflects the expression of two genes predicted to encode multi-copper oxidases and 22 genes encoding putative catalases or peroxidases (Woo et al. 2014). These observations suggest that different *Burkholderia* species utilize different assimilation mechanisms; thus *Burkholderia* sp. strain CCA53 may use a specific pathway for degradation of *p*-hydroxybenzene monomers. Such a degradation pathway for 4-nitrobemzoate, one of the *p*-hydroxybenzene monomers, has been proposed in *B. cepacia* (Peres et al. 2001). To evaluate in detail the capability of *Burkholderia* sp. strain CCA53 for lignin degradation, we now plan to sequence the organism’s entire genome and to implement proteome analysis. We anticipate that these studies will elucidate the lignin utilization pathway of *Burkholderia* sp. strain CCA53.

## Conclusion

In this study, we screened lignin-degrading bacteria and the objective bacterium was isolated from leaf soil. Based on 16S rRNA gene sequencing and phylogenetic analysis, the bacterium was identified as *Burkholderia* sp. strain CCA53. *Burkholderia* sp. strain CCA53 demonstrated growth on alkali lignin, although growth was rather poor. The capability to utilize lignin-associated aromatic monomers was also relatively limited. The most effective utilization was observed for *p*-hydroxybenzene monomers. The results obtained in this study indicate that *Burkholderia* sp. strain CCA53 has fragmentary activity for lignin degradation.
